# Draft Genome Sequences of Pseudomonas sp. Strains MWU13-2100 and MWU13-2105 Isolated from Wild Cranberry Bog Soil in the Cape Cod National Seashore

**DOI:** 10.1128/mra.00557-22

**Published:** 2022-09-22

**Authors:** Brian Mayer, Scott Soby

**Affiliations:** a Biomedical Sciences, College of Graduate Studies, Midwestern University, Glendale, Arizona, USA; b College of Veterinary Medicine, Midwestern University, Glendale, Arizona, USA; SIPBS, University of Strathclyde

## Abstract

Pseudomonas sp. strains MWU13-2100 and MWU13-2105 were isolated from a wild cranberry bog with Pipestone loamy coarse sand soil in Truro, Massachusetts, and taxonomically assigned based on whole-genome sequences. The draft genomes are most closely related to *P. batumici* (41.4% and 41.8% dDDH_d4_), but with only 50.8 dDDH_d4_ to each other.

## ANNOUNCEMENT

Like other wetlands bog soils, cranberry bog soil is a rich microbial environment, yet little is known about interactions between microbes, or with their host plants. Pseudomonas spp. have been implicated in suppression of fungal diseases and maintaining a beneficial environment for plant growth (1–8). Understanding the properties of these bacteria could have potential benefits that could be translated to increasing crop yields and quality. Thus, identifying soil bacteria and their products is important. Pseudomonas sp. strains MWU13-2100 and MWU13-2105 were isolated from wild cranberry bog soil in the Cape Cod National Seashore (42.064742, −70.117562) by briefly vortexing a ≈2g sample from the top 5 cm of the soil profile in sterile water. The supernatant was plated on King’s medium B (KMB) agar containing 50 μg mL^−1^ each of cycloheximide and ampicillin, incubated at 26°C for 48 h, colony-purified 3×, and stored in 34% glycerol at −80°C. Isolates were recovered from frozen storage on fresh KMB, and populations were inoculated into overnight KMB broth cultures. DNeasy blood and tissue kits (Qiagen) were used for gDNA isolation, and the genome was sequenced at the Arizona State University Genomics Core facility. Illumina-compatible genomic DNA (gDNA) libraries were generated with a Kapa Biosystem Hyperplus library preparation kit (KK8514). DNA was enzymatically sheared to ≈500bp fragments, end repaired, and A-tailed. Illumina-compatible adapters with unique indexes (IDT 00989130v2) were individually ligated to each sample, cleaned using pure beads (Kapa Biosciences; KK8002), and amplified with Kapa HiFi enzyme (KK2502). Each library was analyzed for fragment size (Agilent Tapestation) and quantified by qPCR (Kapa library quantification kit, KK4835; Thermo Fisher Scientific, Quantstudio 5) before multiplex-pooling and sequencing on a Illumina MiSeq 2 × 250bp flow cell. Unicycler v0.4.8 (9) was used to assemble the raw reads and Pilon v1.23 (10) was used to polish them in the PATRIC Comprehensive Genome Analysis pipeline v3.6.12. Default parameters were used for all software except for Trim in PATRIC, which was set to “true” (11). Trim Galore v0.4.0 and QUAST v5.1 (12, 13) were used for adapter trimming and quality control Table 1. Genome sequences were annotated using RASTtk v1.073 (14) as part of the PATRIC pipeline. MWU13-2100 and MWU13-2105 were placed taxonomically using the Type (Strain) Genome Server v342 (TYGS; https://tygs.dsmz.de/) (15). The isolates were most similar to *P. batumici* UCM B-321^T^ (JXDG00000000.1) but with only 41.8 and 41.4% dDDH_d4_ values, respectively.

**TABLE 1 tab1:** Data summary for Pseudomonas sp. MWU13-2100 and MWU13-2105

Isolate	Biosample	Genome size (bp)	no. of Contigs	% GC content	Coverage (x)	Total reads (x10^6^)	Total read length (bp)	Read length (bp)	*N*_50_
MWU13-2100	SAMN27107747	6,589,945	178	61.33	105	3.01	692,910,330	229	114,002
MWU13-2105	SAMN27107502	7,183,010	102	61.51	91	2.72	650,339,742	238	281,724

### Data availability.

This whole-genome sequence project has been deposited at DDBJ/EMBL/GenBank under BioProject PRJNA691338 with the accession numbers JALLIX000000000 for MWU13-2100, and JALLIY000000000 for MWU13-2105. The versions described in this paper are JALLIX000000000.1 and JALLIY000000000.1, respectively. Sequence Read Archive (SRA) are available from GenBank under the accession numbers SRR18662642 for MWU13-2100 and SRR18662649 for MWU13-2105. RASTtk annotations are available under open license at Zenodo (https://zenodo.org/record/6399230#.YpZNMqDMKUk and https://zenodo.org/record/6399253#.YpZNCqDMKUk).[Table tab1]
